# Comment on “Evaluation of Manual and Two-Rotary NiTi Retreatment Systems in Removing Gutta-Percha Obturated with Two Root Canal Sealers”

**DOI:** 10.1155/2013/694027

**Published:** 2013-05-27

**Authors:** Seema K. Dixit, Varun Arora, Kapil Loomba, Ashutosh Dixit, Ridhima Birmani Gaunkar, Bhaskar Agarwal, Alok Misra, Narendra kumar Gupta

**Affiliations:** ^1^Department of Endodontics & Conservative Dentistry, Seema Dental College, Rishikesh 249203, India; ^2^Active Research Group, Arun Professional Services, Tulsidas Marg, Chowk, Lucknow 226003, India; ^3^Department of Conservative Dentistry, Saraswati Dental College, Lucknow 227105, India; ^4^Department of Periodontics, Seema Dental College, Rishikesh 249203, India; ^5^Department of Public Health Dentistry, Goa Dental College, Goa 403401, India; ^6^Department of Prosthodontics, Faculty of Dental Sciences, King George Medical University, Lucknow 226003, India; ^7^Department of Endodontics & Conservative Dentistry, Career Institute of Dental Sciences and Hospital, Lucknow 226002, India; ^8^Department of Prosthodontics, Babu Banarasi Das College of Dentistry, BBD University, Lucknow 227015, India

## Abstract

Proper research design, appropriate evaluation and measurement methods, use of correct statistical tests, interpretation, and inference are the essentials without which any scientific research reporting is incomplete and does not serve its proposed purpose. In this communication, some common flaws in research designing, evaluation, analysis, and inference have been explained using a published article as a reference. The purpose of the paper is to help the scientific community recognize the significance of proper research designing and planning in order to achieve the results which are worthwhile.

With reference to the research article entitled “*Evaluation of manual and two-rotary NiTi retreatment systems in removing Gutta-Percha obturated with two root canal sealers*” [1], we would like to point out certain queries related to research design, statistical and inferential errors.

*Scoring Method.* The authors have shown scoring criteria of debris in Section 2.4 under the heading *Analysis*. The criteria seem to be observer dependent. Such methods can be highly subjective in nature and hence are not reliable unless they are proven unanimously. As scores are ordinal in nature and the criteria for calculation being highly subjective, it is difficult to assume that these criteria are replicable unless otherwise proven. The authors have quoted a reference for using the criteria [2]; however, we are sorry to state that the source article too does not justify the use of the criteria as a reliable tool. For observations needing subjective evaluation, reliability and replicability are the most essential requirements [3]. The contemporary literature supports achieving multiple-observer agreement under such situations [4, 5]. Such revalidation is essential as ordinal scoring pattern varies considerably owing to minor change in subjective evaluation. For example, observer 1 might evaluate 49% debris in a case while the same case might be evaluated as 51% by observer 2, thus scoring them as 2 and 3, respectively, which on an ordinal scale with a 5-point scale shows 20% variation in results.

*Statistical Tools.* It is difficult to digest that the authors have used scoring pattern using an ordinal scale (nonparametric in nature) but have analyzed the results using parametric tests (ANOVA and Newman-Keuls test). Even if they have used parametric test (ANOVA), choice of Newman-Keuls test seems to be inappropriate as it tends to increase the Type I error. Although the whole point of multiple comparison post hoc tests is to keep the chance of a Type I error in any comparison to be 5%, in fact the Newman-Keuls test does not do this. In some cases, the chance of a Type I error can be greater than 5%. (The Newman-Keuls test works fine with three groups; the increase in Type I error occurs only with four or more groups.) Because the Newman-Keuls test works in a sequential fashion, it cannot produce 95% confidence intervals for each difference. In contrast, other post hoc tests, namely the Tukey test, can compute confidence intervals [6].

*Incomplete Representation.* In Table 2 of the article in question, time necessary for retreatment of different groups has been shown as a mean value. This is not consistent with Table 1, where all the values have been shown as mean ± SD. In the absence of representation of standard deviation in this table it is difficult for the reader to estimate the extent of sample-to-sample variability within group. With this incomplete picture, the authors have concluded that two-rotary techniques have consumed significantly less time in retreatment. However, no statistical substantiation of the same has been done. For that matter, outcome of statistical results has not been shown in either of the two tables.

*Missing Retreatment Method for Group 4.* In Methodology section, the authors describe endodontic retreatment method for Groups 1 and 3, Groups 2 and 5, and Groups 3 and 6 (here Group 3 appears twice, and Group 4 is missing, so it is difficult to comprehend which technique is proposed for Group 3 and which one for Group 4); thus making Group 3 appear twice and missing Group 4, this is a very serious flaw given the complex study design using too many combinations of initial endodontic treatment, instrumentations, and retreatment methods.

Under these circumstances, we feel that the inferences drawn in the said article need a clarification.
